# BLNCR is a long non-coding RNA adjacent to integrin beta-1 that is rapidly lost during epidermal progenitor cell differentiation

**DOI:** 10.1038/s41598-018-37251-w

**Published:** 2019-01-10

**Authors:** Sabine E. J. Tanis, Elif Senem Köksal, Jessie A. G. L. van Buggenum, Klaas W. Mulder

**Affiliations:** Radboud University, Faculty of Science, Radboud Institute for Molecular Life Sciences, Department of Molecular Developmental Biology, Nijmegen, The Netherlands

## Abstract

As our understanding of transcriptional regulation improves so does our appreciation of its complexity. Both coding and (long) non-coding RNAs provide cells with multiple levels of control and thereby flexibility to adapt gene expression to the environment. However, few long non-coding RNAs (lncRNAs) have been studied in human epidermal stem cells. Here, we characterized the expression of 26 lncRNAs in human epidermal keratinocytes, 7 of which we found to be dynamically expressed during differentiation. We performed in depth analysis of a lncRNA located proximal to the epidermal stem cell marker integrin beta-1 (ITGB1) and transcribed in the opposite direction. We dubbed this gene Beta1-adjacent long non-coding RNA, or BLNCR, and found that its expression is regulated by p63 and AP1 transcription factors. Furthermore, BLNCR expression is regulated downstream the integrin and EGF signaling pathways that are key to epidermal stem cell maintenance. Finally, we found that BLNCR expression is rapidly reduced upon induction of differentiation, preceding the down regulation of integrin beta-1 expression. These dynamics closely mirror the loss of proliferative and adhesion capacity of epidermal stem cells in colony formation assays. Together, these results suggest that loss of BLNCR expression marks the switch from a proliferative state towards terminal differentiation in human epidermal stem cells.

## Introduction

Although historically research has predominantly focused on protein encoding genes, it is now evident that non-coding RNAs play important roles in the regulation of many, if not all, biological processes. MicroRNAs are a well-studied class of non-coding RNAs that is involved in regulation of transcript levels and translation of mRNAs. Less clearly defined are the various functions of long non-coding RNAs (>200 nucleotides, lncRNA), which are almost as numerous as protein encoding transcripts in number of annotated genes in the human genome^1^. LncRNAs have been implicated in many developmental processes ranging from dosage compensation (e.g. Xist), imprinting (e.g. Kcnq1, Airn), neuronal development (e.g. Peril, Evf) and stem cell differentiation of neuronal, cardiac, epidermal, endodermal, endothelial and hematopoietic cells^2^. In addition, their involvement in various types of cancer and other pathologies highlight the importance of identifying and characterizing these transcripts^3^. Diverse modes of action have been identified for lncRNAs, including functioning as a decoy preventing protein-DNA interactions, a scaffold for the assembly of protein complexes (e.g. HOTAIR) or a guide leading protein complexes to specific DNA sequences^4–7^. Recently, it has been shown that the transcriptional activity of lncRNA promoters can regulate transcription of neighboring genes in *cis* and that such regulation is not necessarily restricted to lncRNA genes^8^.

The versatility of the mechanisms through which lncRNAs contribute to the regulation of biological processes provides the cells with yet another layer of (post-) transcriptional control. In light of this, we are particularly interested in their role in stem/progenitor cell differentiation in the epidermal layer of the skin. The high renewal rate of the epidermal layer requires tight regulation of gene expression programs governing proliferation and differentiation in human epidermal stem cells. These cells are anchored to the basement membrane by integrin subunits and are released upon initiation of differentiation, enabling them to traverse several stages of differentiation to eventually be sloughed from the surface of the epidermis. During these transitions, many genes are dynamically expressed to play regulatory roles, for instance directly influencing transcription programs (transcription factors), indirectly via signaling pathways (EGF, Notch, adhesion) or through (post-)transcriptional regulation (ncRNAs). To date, only a few lncRNAs have been implicated in keratinocyte biology, with ANCR being the first well-defined one. This lncRNA was found to be necessary for the maintenance of the undifferentiated state via the regulation of differentiation genes across the genome^9^. Another lncRNA identified by the same group, TINCR, was shown to be required for epidermal differentiation by its involvement in stabilizing mRNA transcripts from differentiation genes^10^.

Here, we validated the expression of 26 lncRNAs identified by the GENCODE consortium, and found that several of these genes are differentially expressed during keratinocyte differentiation. Although some are most likely enhancer RNAs, most have their own promoters that are decorated with active histone marks as well as RNA polymerase II activity, indicating that they are independently transcribed genes. Furthermore, we characterized a lncRNA that is immediately adjacent to ITGB1 and named it Beta1-adjacent-lncRNA (BLNCR). We found that ITGB1 and BLNCR are closely spaced genes that are transcribed from opposite strands of the DNA and that the p63 and AP-1 transcription factors are involved in regulating their expression. We also investigated the contribution of signaling pathways, where inhibition of MEK and knock-down of ITGB1 indicated that both EGF and integrin signaling impact BLNCR and ITGB1 expression. Interestingly, BLNCR expression seems more attuned to MEK mediated EGF signaling, possibly via an AP-1 mediated enhancer interaction. The timing of down regulation suggests that BLNCR is involved in the earlier events towards differentiation as it coincides with the point of no return in proliferation arrest while preceding the loss of adhesion capacity in colony formation assays. Together, we identified BLNCR as a new epidermal progenitor cell marker that is rapidly down regulated upon induction of differentiation, potentially marking early events of epidermal stem cell differentiation.

## Methods

### Cell culture

Human primary keratinocytes from two different body sites (LKA; oral and KNP; foreskin, obtained from Lonza Ltd.) were cultured on an mitotically inactivated layer of J2-3T3 fibroblast as described before^11^. Prior to induction of differentiation, the cells were grown feeder-free in Keratinocyte Serum-Free Medium (KSFM, Gibco) supplemented with 30 μg/mL bovine pituitary extract (BPE, Gibco) and 0.2 ng/mL epidermal growth factor (EGF, Gibco). Colony formation assays were performed for seven days on inactivated feeders after AG1478 treatment on KSFM for the indicated times.

### Induction of differentiation and kinase inhibition

Differentiation was induced using 10 μM EGFR inhibitor AG1478 (Calbiochem) and/or 200 ng/mL human recombinant BMP 2/7 (R&D systems) in KSFM for the time indicated in the figure legends. In the kinase-inhibitor experiments MEK inhibitor PD0325901 (MEKi, Axon Medchem) was used at a concentration of 1 μM for the indicated time.

### Nucleofection of siRNAs

Keratinocytes were grown to 70% confluency in KSFM, where after they were harvested, counted and 200.000 cells were mixed with siRNA 2 μM final in a 20 μL transfection reaction. Nucleofection was performed using the Amaxa 4D 96 well nucleofector (program FF113). The cells were rested for 10 minutes before seeding.

### RNA extraction and RT-qPCR

Prior to RNA extraction cells were washed once with PBS and lysed using RNA lysis buffer provided with the RNA extraction kit. RNA extraction was performed using the Quick RNA Microprep kit (Zymo Research) according to manufacturer’s protocol. Reverse transcription was performed using Maxima RT enzyme (Thermo Fisher Scientific) according to manufacturer’s protocol followed by qPCR. In short, RNA was annealed with 0,5 µg/µL hexamer primers in the presence of 0.5 mM dNTPs for 5 minutes at 65 °C, where after the reverse transcriptase reaction was performed using maxima RT enzyme and RNase inhibitor RNasin plus. Samples were incubated 10 minutes at 25 °C, 45 minutes at 50 °C and finally 5 minutes at 80 °C. Samples were treated with RNaseH (2U per sample) for 20 minutes at 37 °C before being diluted to 5 ng/μL for qPCR. qPCR reactions of 20 μL contained 10 ng cDNA, mixed with 10 μL 2x SYBR green Supermix (Biorad), 5 pmol forward primer, 5 pmol reverse primers and MQ to 20 μL. A standard qPCR protocol was run (3 min 95 °C, [10 sec. 95 °C, 30 sec. 60 °C] × 39) and data were analyzed in Excel.

### Publicly available ChIP sequencing data and RNA sequencing data

Previously published p63, RNA polymerase II and H3K27Ac ChIP sequencing tracks were used from Kouwenhoven *et al*., EMBO Reports, 2015, 16(7):863-78^12^. Publicly available RNA sequencing tracks were used from Kretz, M. *et al*., 2012 (GSE35468)^9^.

### Colony formation assays

Cells were cultured as described under “cell culture”, harvested and put on KSFM for AG1478 treatment. After the indicated times the cells were harvested and passaged onto inactivated J2-3T3 fibroblasts. After 7 days, the fibroblasts were washed away and the remaining colonies were fixed using 4% formaldehyde. Cells were permeabilized using 0.1% Triton. The cells were blocked and stained in PBS + 10% serum with DRAQ5 (1:4000, Biostatus) and anti-TGM1. DRAQ5 signal was measured in Image Studio V5.0 using the Odyssey CLx system and colonies number and size was determined using CellProfiler. Data analysis and visualisation was performed in R.

### 5′ RACE

Performed as described in Scotto-Lavino *et al*.^13^, [17406509]. In short, the 5′ end of BLNCR was targeted in a primed RT reaction and after polyadenylation of the cDNA a universal anchor was attached. This was followed by a nested PCR strategy amplifying the 5′ end of the cDNA specifically (see Table 1 for primer sequences). The PCR products are were purified using an TA cloning strategy (source) and the the 5′-end determined using Sanger sequencing.

**Table 1:** Primers (5′-3′).

Name in figure	Sequence
RT	CCCAGGCACAGAGAAATGGT
GSP1	CACATCTGGCAGGGTGTCTT
GSP4	CTTCGCTTAAGGTCCCCCTG
GSP5	TGGCTGCTGGTGACAAAGAA

## Results

### Expression of 26 lncRNAs in proliferating and differentiated epidermal keratinocytes

**Figure 1:**
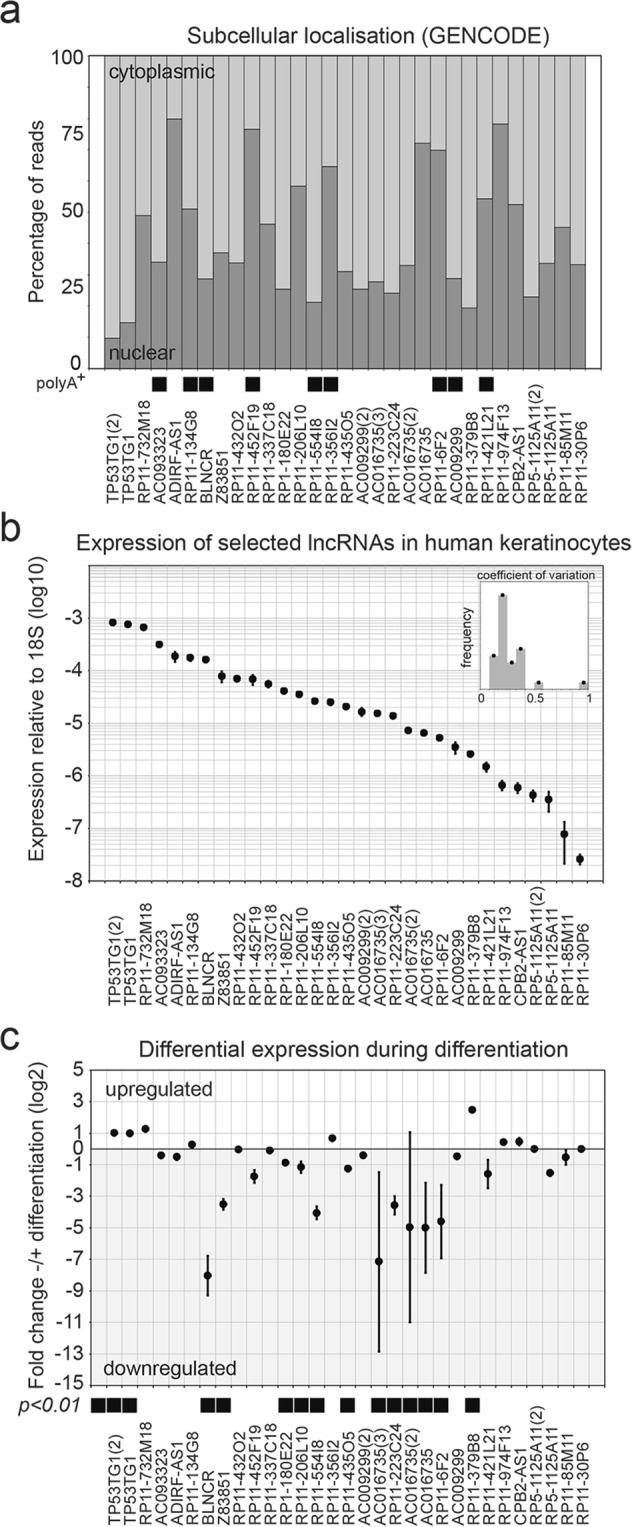
Selected lncRNAs are expressed in self-renewing and differentiating keratinocytes. (**a**) Percentage of reads with cytoplasmic or nuclear localization of selected lncRNAs. PolyAdenylated lncRNAs are indicated with black squares. Numbers between brackets indicate the number of primer sets used in panel b and c. (**b**)Expression of selected lncRNAs in self-renewing keratinocytes. The expression is normalized based on 18s expression. n = 3, mean −/+ SD. The small inset graph shows that the coefficient of variation is generally below 30%. (**c**) Fold change differentially expressed lncRNAs after 48 hours AG1478 mediated EGFR inhibition. n = 3, mean −/+ SD.

The function of only a few lncRNAs have been studied and characterized in primary human epidermal stem cells (keratinocytes)^9,10^. Of these, ANCR and TINCR were found to be dynamically expressed and are important for self-renewal and differentiation, respectively^9,10^. To explore other lncRNAs that may be functionally relevant in this system, we selected 26 lncRNAs that were identified in primary human keratinocytes in the context of the GENCODE consortium^1^. Our selection covered both polyAdenylated and non-polyAdenylated transcripts, as well as lncRNAs that were (predominantly) nuclear or cytoplasmic in their localization (Fig. 1a). We found a broad dynamic range of expression for these transcripts, covering more than three orders of magnitude (Fig. 1b). The low coefficient of variation (triplicates, generally <50%, Fig. 1b, inset) indicated that even the low expressed lncRNAs were reproducibly and reliably detected. We confirmed the accuracy of detection for several of the transcripts across the range of expression levels using multiple independent qPCR primer pairs (Fig. 1b, numbers between brackets).

**Figure 2:**
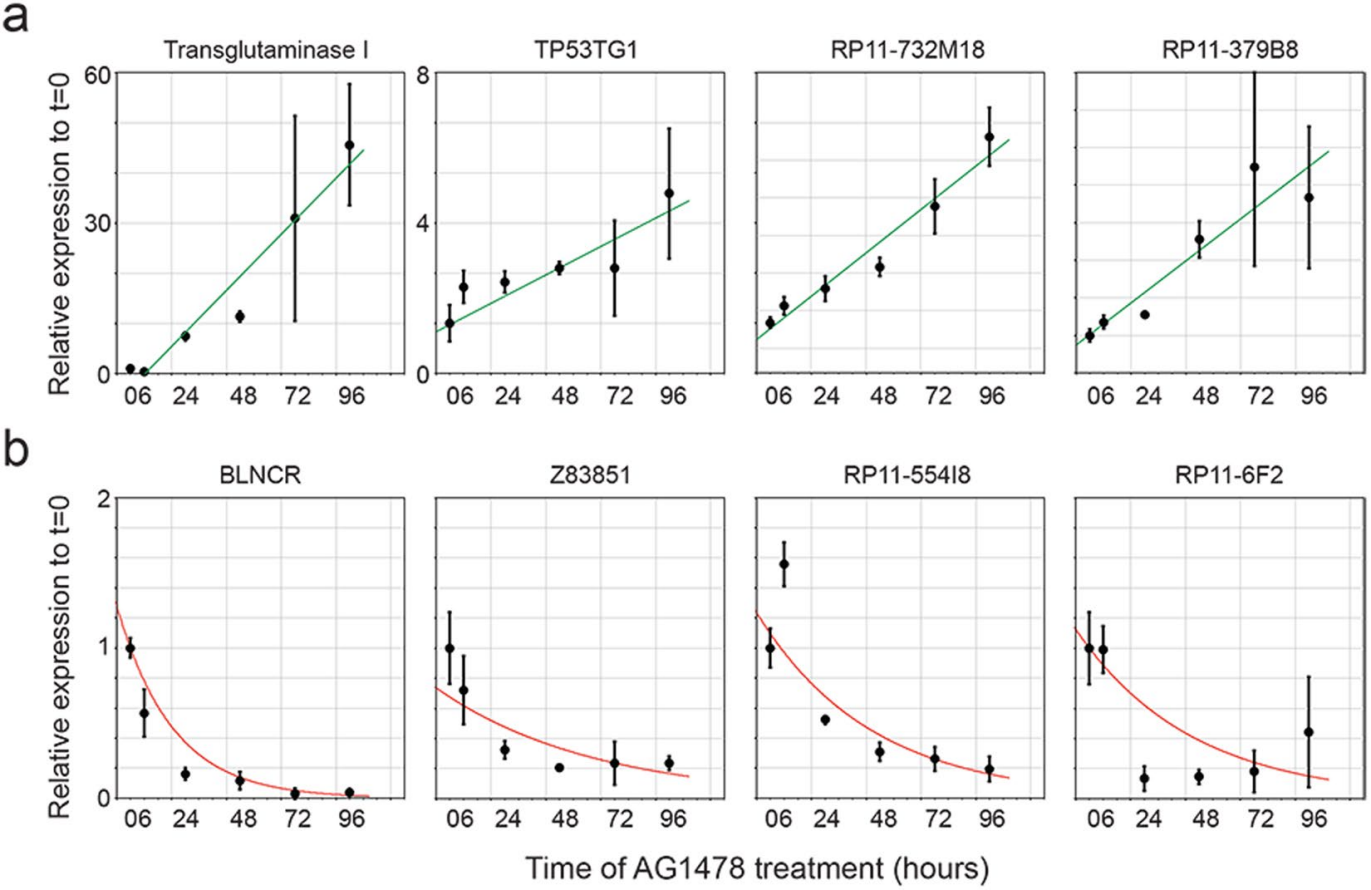
Time-course induction of differentiation using AG1478 shows different expression kinetics. (**a**)Expression dynamics of upregulated terminal differentiation marker TGM1 and lncRNAs TP53TG1, RP11-732M18 and RP11-379B8 over time. Expression is shown relative to T = 0. n = 3 (**b**) Expression dynamics of downregulated lncRNAs BLNCR, Z83851, RP11-554I8 and RP11-6F2 over time. The expression is shown relative to T = 0. n = 3, mean −/+ SD.

After corroborating the expression of the lncRNAs in undifferentiated keratinocytes, we wanted to know if they were differentially expressed during differentiation. When we induced differentiation using the EGFR inhibitor AG1478 for 48 hours^14^, we noted that several lncRNAs exhibited pronounced differential expression upon differentiation (Fig. 1c). We choose seven lncRNAs for further characterization based on their strong expression dynamics with low variation upon induction of differentiation, as this might indicate that they are involved in this process. To evaluate their expression kinetics, we performed a time-course of AG1478 induced keratinocyte differentiation, extracting mRNA at 0, 6, 24, 48, 72 and 96 hours after treatment. Strong activation of the late differentiation marker transglutaminase I confirmed the induction of differentiation in our assay (Fig. 2a). All seven lncRNAs showed differential expression concordant with the previous results (Figs 1c and 2a,b). Moreover, this experiment revealed that lncRNAs TP53TG1, RP11-732M18 and RP11-379B8 were up regulated during differentiation with comparable kinetics (Fig. 2a). In contrast, the four down regulated transcripts (BLNCR, Z83851, RP11-554I8 and RP11-6F2) exhibited different types of kinetics, especially at the early (6 and 24 hour) time-points (Fig. 2b). Together, our analysis highlighted seven lncRNAs that are differentially expressed during keratinocyte differentiation, suggesting that they may be linked to this process.

### ITGB1 and BLNCR are closely spaced neighboring genes transcribed in opposite directions

We wondered whether the expression of these lncRNAs was a consequence of transcriptional activity of neighboring loci or if these genes are independently transcribed^8,15^. We confirmed expression of the lncRNAs and their neighboring genes using an independent public dataset on RNA expression on day 0, 3 and 6 of differentiation^9^. We then mined publicly available epigenomic profiling datasets to see if there were indications of histone modifications associated with active transcription and/or enhancer activity at the dynamically expressed lncRNA loci. This analysis supported the notion that RP11-554I8 and RP11-379B8 were genes driven by their own independent promoters. Even though these genes seemed to be embedded in active enhancer regions (as identified by the H3K4me1 and H3K27ac histone modifications) of adjacent genes, the presence of the active promoter associated H3K4me3 modification, as well as RNA polymerase II, shows that these lncRNAs are independent transcription units (*up*. Fig. 1a,b *upper panels*). Moreover, published RNA-sequencing data and genome annotation data from the GENCODE and HAVANA projects^1^ revealed distinct intron-exon structures for these genes, arguing against these transcripts being enhancer-derived RNAs (data not shown). In contrast, the putative lncRNA Z83851 is located just upstream of the known lncRNA LOC388906 in a region which does not contain H3K4me3, but does harbor H3K4me1 and H3K27ac signal, indicating that it is likely to be an enhancer RNA (Sup. Fig. 1c upper panel).

**Figure 3:**
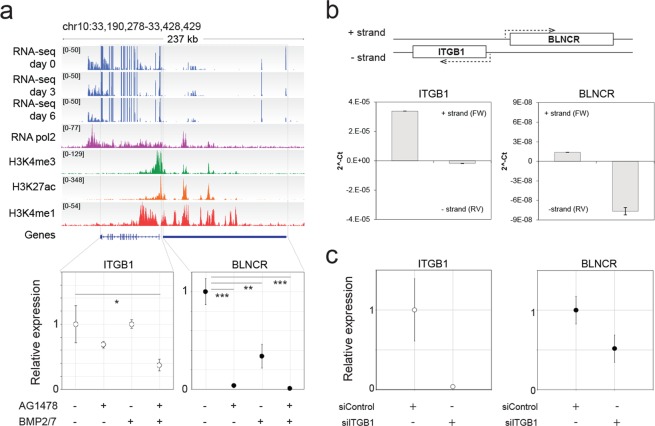
lncRNA gene BLNCR is located nearby epidermal stem cell marker ITGB1 and is transcribed independently. (**a**) Upper panel: Publicly available data of RNA-sequencing show expression of both transcripts (Kretz, M. *et al*., 2012 (GSE35468)) and histone marks (NHEK tracks, ENCODE). Lower panels: Expression of ITGB1 and BLNCR during differentiation. n = 3, mean −/+ SD. Statistical significance was assessed using a t-test: *p < 0,05; **p < 0,01; ***p < 0.001. (**b**) Detection of RT-product by qPCR of ITGB1 and BLNCR after strand-specific RT-qPCR. n = 3, mean −/+ SD. (**c**) Relative expression of ITGB1 and BLNCR after 48 h knockdown of ITGB1. Percentage knockdown ITGB1: 96%, n = 3, mean −/+ SD. Statistical significance was assessed using a t-test: *p < 0,05; **p < 0,01; ***p < 0.001.

As some lncRNAs regulate transcription of other genes *in cis*^8^, we investigated whether genes that were immediately adjacent to the identified lncRNA genes were also differentially expressed. Using our previously published expression profiling data on keratinocytes treated with vehicle, AG1478, BMP 2/7, or a combination of these compounds^14^, we assessed the expression of genes close to BLNCR, RP11-554I8, RP11-379B8 and Z83851. In addition, we measured the expression of the non-coding RNAs under the same conditions by RT-qPCR for comparison (bottom panels of Fig. 3a, Sup. Fig. 1). We found that these four non-coding RNAs are indeed differentially expressed under these conditions, consistent with the results presented in Figs 1 and 2. Moreover, the genes adjacent to BLNCR and RP11-379B8 were also differentially expressed (Fig. 3a, Sup. Fig. 1).

The gene proximal to BLNCR is of particular interest, as it encodes the key epidermal progenitor marker integrin beta-1 (ITGB1/CD29). This cell surface protein is important for epidermal adhesion to the underlying basement membrane and is a commonly used marker to identify, and select for, human epidermal stem cells in culture and *in vivo*^16^. To validate that these genes are indeed downstream of EGF signaling, we treated the cells with AG1478 and a MEK inhibitor for 24 hours. MEK is a protein kinase that works downstream of the activated EGF receptor in the Ras-Raf-MEK-ERK pathway. We found a clear reduction in ITGB1 and BLNCR expression, indicating that these genes are indeed downstream of EGF signaling (Sup. Fig. 2). Further exploration indicated that the ITGB1 and BLNCR transcription start sites are located near each other (~500 bp apart, Sup. Fig. 3), which we subsequently confirmed using 5′-RACE (Sup. Fig. 4). This suggests that these genes potentially share their promoter region. Additionally, BLNCR is likely not an enhancer derived RNA as it is spliced post-transcriptionally (Sup. Fig. 3). Finally, we established that ITGB1 and BLNCR are indeed transcribed from opposite strands of the DNA template using strand-specific RT-qPCR analysis (Fig. 3b).

There are several ways via which lncRNAs can regulate transcription of genes in *cis*, including transcript specific mechanisms. To evaluate if expression of BLNCR is dependent on the presence of the ITGB1 transcript, we knocked-down ITGB1 mRNA levels using siRNAs. We found that ITGB1 knock-down consistently reduces the expression of BLNCR about two-fold (Fig. 3c). This relatively mild effect on BLNCR levels, notwithstanding a very efficient ITGB1 knock-down, suggests that this reduction is likely to be an effect of impaired integrin-mediated signaling, rather than *in cis* transcriptional regulation. Despite repeated and extensive attempts, we were unable to obtain efficient (>50%) silencing of the BLNCR lncRNA and were therefore not able to conclusively investigate *cis*-regulation of ITGB1 expression by the adjacent lncRNA.

### P63 and AP-1 transcription factors regulate BLNCR and ITGB1 expression

**Figure 4:**
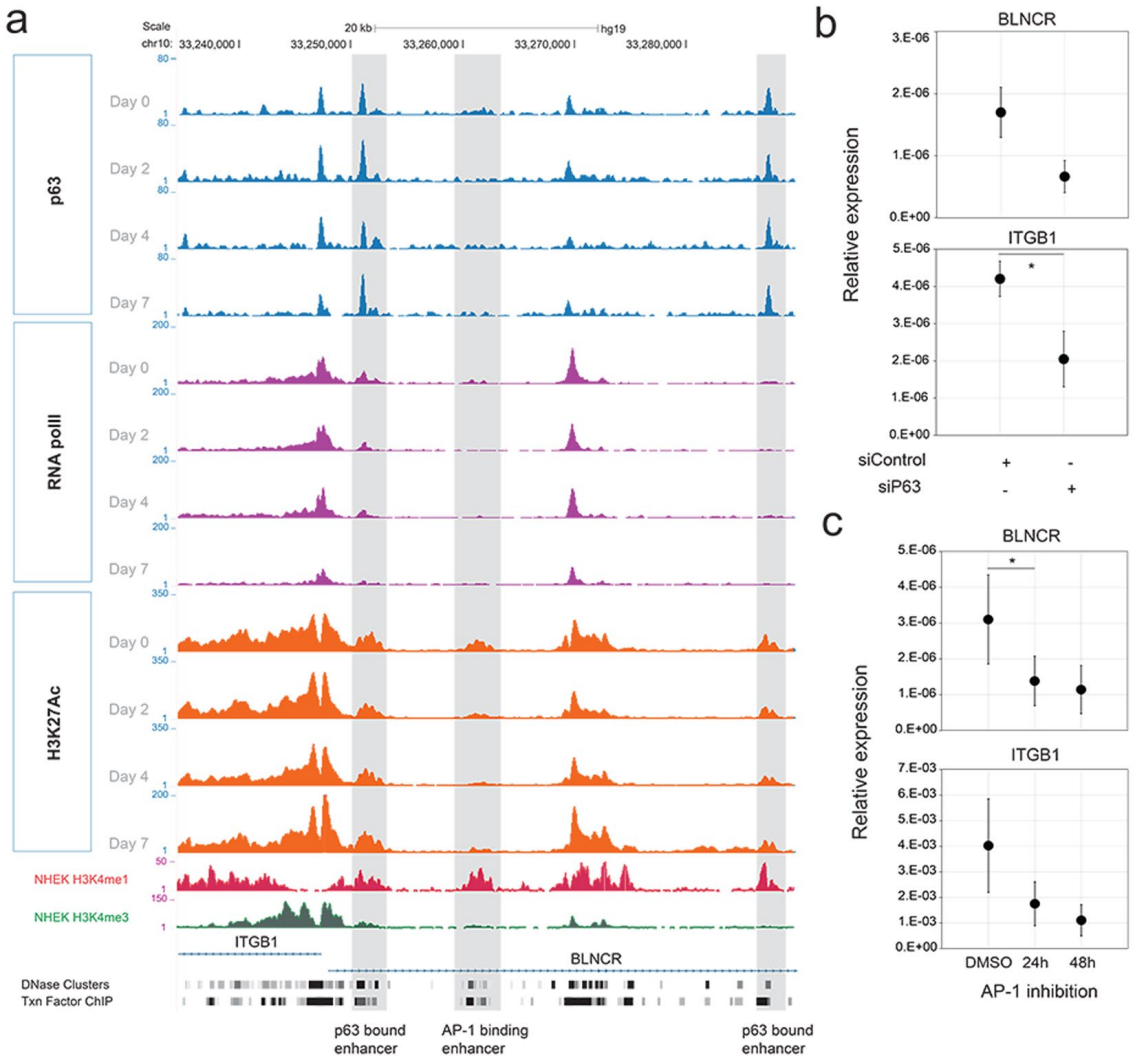
Expression of ITGB1 and BLNCR is regulated by p63 and AP-1 factors. (**a**) ChIP-sequencing tracks of p63, RNA pol II and H3K27ac signal in promoter region of BLNCR and ITGβ1 during a time-course of differentiation; day 0, 2, 4 and 7^12^. Non-differentiated NHEK H3K4me3 and H3K4me1 tracks from ENCODE, DNase clusters (ENCODE, 125 cell types) and transcription factor ChIP-seq tracks (ENCODE transcription factor ChIP-seq, 161 factors, with Factorbook Motifs) are also included. (**b**) 18s normalized expression of indicated genes in p63 knockdown samples. Percentage knockdown p63: 82,5%. n = 3, mean −/+ SD. Statistical significance was assessed using a t-test: *p < 0,05; **p < 0,01; ***p < 0.001. (**c**) 18s normalized expression of indicated genes after 24 or 48 hours of AP-1 inhibition using tanshinone IIA. n = 3, mean −/+ SD. Statistical significance was assessed using a t-test: *p < 0,05; **p < 0,01; ***p < 0.001.

Next, we set out to explore potential DNA binding transcription factors involved in the regulation of ITGB1 and BLNCR expression. P63 is a key transcription factor essential for skin development and plays a pivotal role in both keratinocyte proliferation and differentiation^17^. We noticed that p63 binds to a proximal and a distal enhancer region of ITGB1 and BLNCR in a publicly available dataset on a time-course of keratinocyte differentiation^12^ (Fig. 4a). This dataset covers p63, RNA-polymerase II, H3K27ac ChIP-seq experiments for day 0, 2, 4 and 7 of growth factor deprived and confluency induced, differentiated keratinocytes. We confirmed that expression of both ITGB1 and BLNCR was strongly down regulated in this differentiation system using RT-qPCR (Sup. Fig. 5).

To assess if p63 is indeed involved in the regulation of BLNCR and ITGB1 we knocked down p63 in proliferating primary keratinocytes (Sup. Fig. 6a). In absence of p63, the expression of both BLNCR and ITGB1 is down regulated, as is the expression of other stem cell- and differentiation- markers (ITGA6, PPL, and TGM1, Fig. 4b, Sup. Fig. 6). This suggests that p63 is involved in the transcriptional regulation of ITGB1 and BLNCR, although we cannot formally exclude the possibility that these results may arise from an effect of cell state in general by silencing p63. Furthermore, the binding of p63 to this enhancer region does not change during differentiation, whereas the expression of ITGB1 and BLNCR does. This is in line with the previously described role of p63 as a bookmarker of genes important for epidermal development^12^. Interestingly, in the timespan that ITGB1 and BLNCR are down regulated in differentiation, RNA polymerase II remains bound their promoter, indicating that the enzyme is possibly stalled in an inactive state^18^. The discrepancy between the down regulation of these genes during differentiation and the retention of p63 binding to their proximal and distal enhancer elements (Figs 2b and 4a), suggests that there might be additional transcription factors or mechanisms involved.

To search for other transcription factors possibly regulating expression of ITGB1 and BLNCR, we looked for dynamics in promoter and enhancer marks on and around the genes and transcription factors binding there. We identified a peak in the H3K27ac signal in the second intron of BLNCR that disappeared decreased during differentiation (Fig. 4a). The absence of H3K4me3 and presence of H3K4me1 indicates that this region functions as a dynamic enhancer (Fig. 4a). As both the BLNCR and the ITGB1 gene are located in the same topologically associated domain (TAD), such a regulatory interaction is likely to occur and affect one, or both, of these genes (Sup. Fig. 7a). Unfortunately, the close proximity of the ITGB1 and BLNCR promoter to this enhancer precludes reliable chromosome conformation capture (3C or 4C) experiments due to high local background signals. Instead, we looked at transcription factor binding hotspots (ENCODE transcription factor ChIP-seq, 161 factors, with Factorbook Motifs) in a DNase I cluster (ENCODE, 125 cell types) in this dynamic enhancer region and found several motifs of transcription factors that  are expressed in our keratinocytes (Sup. Fig. 7b). Our interest was sparked by binding of several AP-1 transcription factor family members as they are known downstream effectors of EGF and integrin signaling, two important renewal signals in keratinocyte biology^19^. We found that treating cells with the AP-1 family inhibitor tanshinone II^20^ resulted in decreased expression of both BLNCR and ITGB1 (Fig. 4c). Together, our experiments suggest that both BLNCR and ITGB1 are, at least in part, regulated by p63 and AP-1 family transcription factors.

### BLNCR expression mirrors loss of proliferative capacity during initiation of differentiation

**Figure 5:**
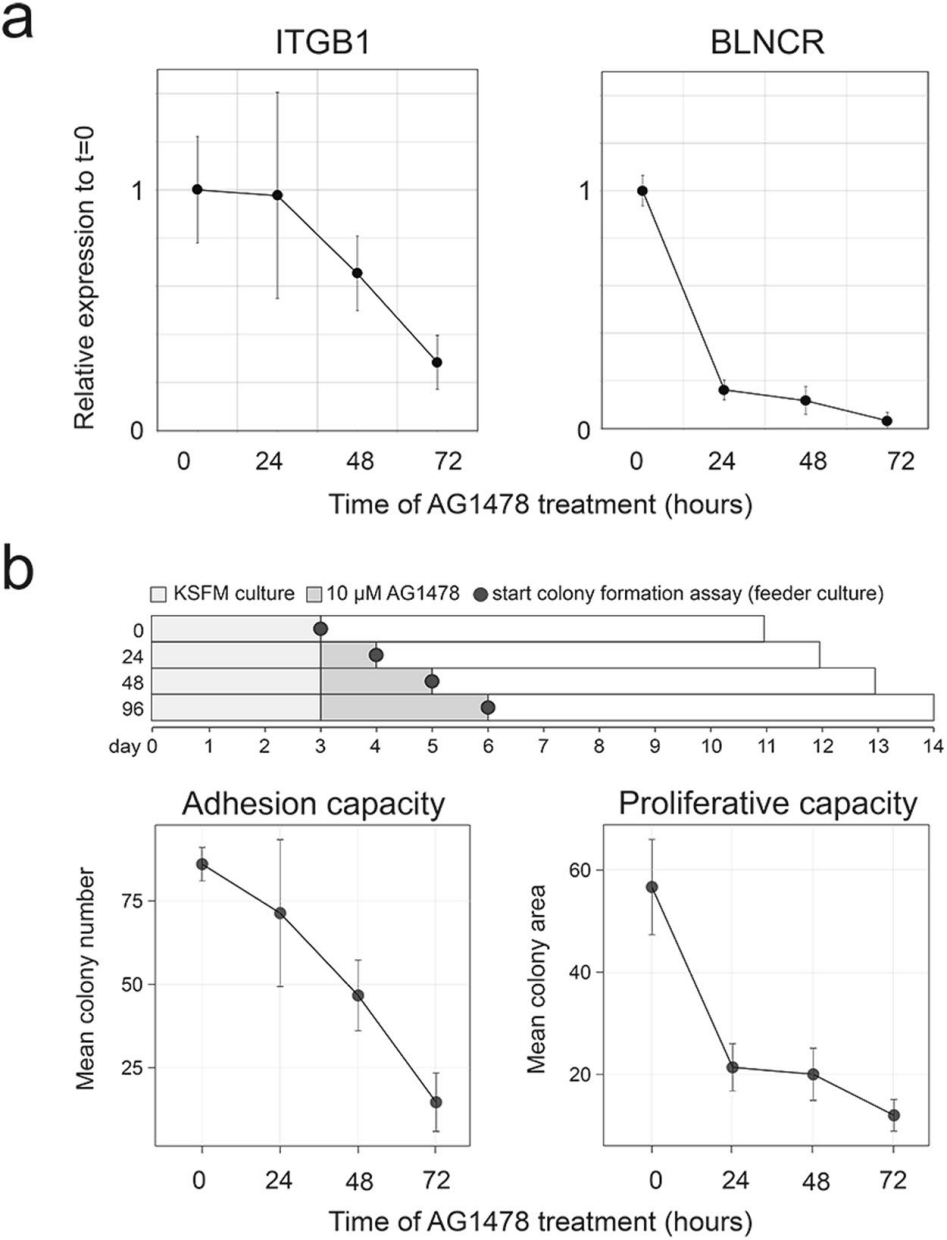
BLNCR expression is regulated by EGF signaling and downregulation precedes the point-of-no-return in differentiation. (**a**) Relative 18s normalized expression of ITGB1 and BLNCR in a time-course experiment with AG1478 treatment. Data on BLNCR is already visualized in Fig. 2. n = 3, mean −/+ SD. Statistical significance was assessed using a t-test: *p < 0,05; **p < 0,01; ***p < 0.001. (**b**) Upper panel: Overview of the assay set-up. Lower panels: Number of colonies and colony area. Cells were treated with 10 μM AG1478 for the indicated times before they were seeded in the CFA. n = 3.

EGF receptor inhibition with AG1478 induces keratinocyte differentiation *in vitro*, recapitulating the process in the epidermis where the cells stop proliferating before releasing their integrin anchors and embarking on their differentiation journey^14^. To understand the relative contribution of ITGB1 and BLNCR regulation with respect to these processes, we first compared the dynamics of ITGB1 and BLNCR down regulation in a time-course of AG1478 induced differentiation. As shown in Fig. 2b as well, BLNCR was essentially entirely down regulated within the first 24 hours (Fig. 5a). In contrast, ITGB1 mRNA levels started to decrease between 24 and 48 hours of treatment. Thus, regulation of BLNCR responds to the differentiation signals earlier than ITGB1. To see if we could put the expression dynamics of BLNCR in the context of proliferation and adhesion capacity of the cells, we used a colony formation assay set-up (Fig. 5b). We treated primary keratinocytes for 0, 24, 48 or 72 hours with AG1478 in defined serum-free medium, whereafter they were seeded on inactivated feeders under standard colony forming assay conditions (without AG1478)^13^. This allowed us to assess at which time point after induction of differentiation the cells will lose their adhesion and proliferative capacity, respectively. This revealed that dynamics of down regulation of ITGB1 expression mirrors the potential of the cells to adhere in the colony formation assay (as monitored by quantifying the *number* of colonies), which is starting to decrease after 24 hours of AG1478 treatment (Fig. 5b, left panel). In contrast, the proliferative capacity of the cells (as monitored by the *size* of the colonies that are formed) is essentially entirely lost within the first 24 hours of AG1478 treatment (Fig. 5b, right panel) and very closely follows the dynamics of down regulation of BLNCR. This observation suggests that loss of BLNCR expression may be an early event in epidermal differentiation and could be associated with the loss of proliferative potential of epidermal stem cells committed to differentiate.

## Discussion

**Figure 6:**
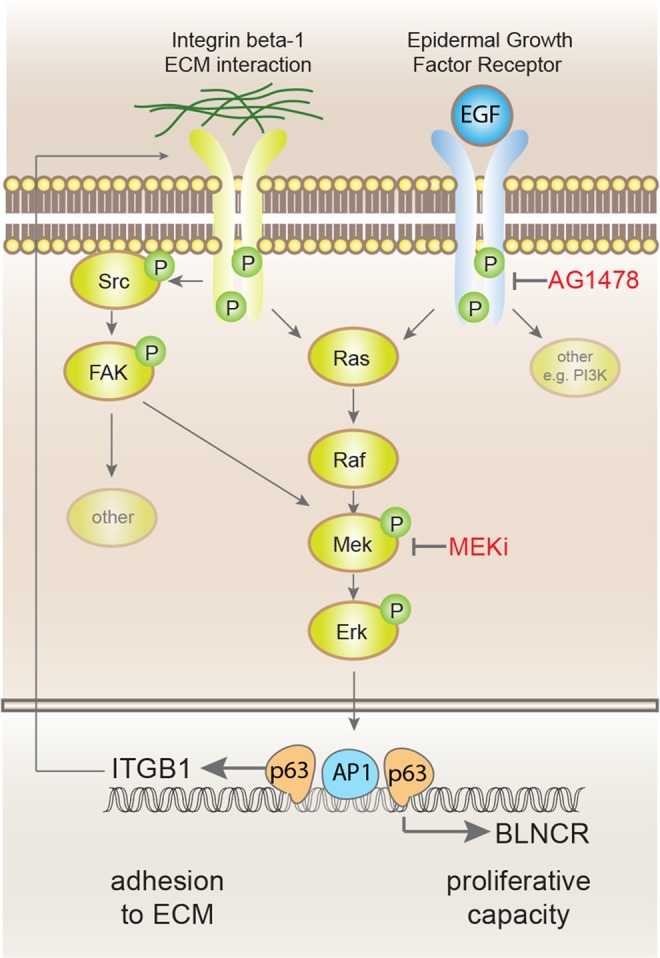
Summarising overview of transcription factors and pathways that regulate expression of BLNCR and ITGB1.

In this work, we validated the expression of 26 lncRNAs in keratinocytes and showed that seven were dynamically expressed during differentiation. BLNCR caught our attention because of its proximity to ITGB1, a critical stem cell marker. Although we could not conclusively investigate *cis*-regulation, we discovered that the expression of both ITGB1 and BLNCR is, at least in part, regulated by p63 and AP-1 factors binding to a presumed shared enhancer region. It seems plausible that BLNCR and ITGB1 are co-regulated to a degree, especially considering their proximity and localization in the same TAD. This could be through the transcriptional activity around a shared promoter region, which can increase local concentration of transcription machinery^8^. In line with this, the expression of both genes involves p63 and AP-1 factors as well as input from the epidermal growth factor and integrin mediated adhesion signaling pathways (summarized in Fig. 6). Interestingly, neither p63 nor RNA-polymerase II binding to BLNCR and ITGB1 seems to be dynamic in the early stages of confluency induced differentiation, whereas the expression of both genes does decrease. For p63, it has been described that it can also function as bookmarker, marking genes important for epidermal development without binding dynamics^12^. RNA polymerase II binding might be unchanging in the early stages of differentiation because its activity might be stalled and remains bound in an inactive state^18^.

The responsiveness of BLNCR expression to EGF and adhesion signalling makes BLNCR an interesting marker that could be used to monitor the proliferative state of the cell. The interplay between these pathways is essential in maintaining a proliferative population, as cells do not proliferate in absence of adhesion even in the presence of growth factors and just adhesion is not enough to trigger proliferation^19^. Using colony formation assays we showed that although AG1478 treated keratinocytes still display normal adhesion efficiency after 24 hours of treatment, their long-term proliferative capacity is already lost. This is also recapitulated in the more rapid down regulation of BLNCR in case of EGF inhibition compared to ITGB1. As the expression of BLNCR is also dramatically reduced in the first 24 hours of differentiation, its expression is potentially associated with the proliferative potential of epidermal stem cells and suggests that the down regulation of BLNCR is an early event leading up to initiation of terminal differentiation.

## Supplementary information

Supplementary Information
