# Supplementary material for: BLNCR is a long non-coding RNA adjacent to integrin beta-1 that is rapidly lost during epidermal progenitor cell differentiation
Source: Sci Rep. 2019 Jan 10;9:31. doi: 10.1038/s41598-018-37251-w (PMC6328539; doi:10.1038/s41598-018-37251-w)
Supplement: Supplementary file 1 — Supplementary Information [file 41598_2018_37251_MOESM1_ESM.pdf]

Supplementary Information

BLNCR is a long non-coding RNA adjacent to integrin beta-1 that is rapidly lost during epidermal progenitor cell differentiation.

Sabine E.J. Tanis[1,*], Elif Senem Köksal[1,*], Jessie A. G. L. van Buggenum[1], Klaas W. Mulder[1,2]

[1]Radboud University, Faculty of Science, Radboud Institute for Molecular Life Sciences, Department of Molecular Developmental Biology, Nijmegen, the Netherlands.

[*]These authors contributed equally to this work

[2]Corresponding author: k.mulder@science.ru.nl

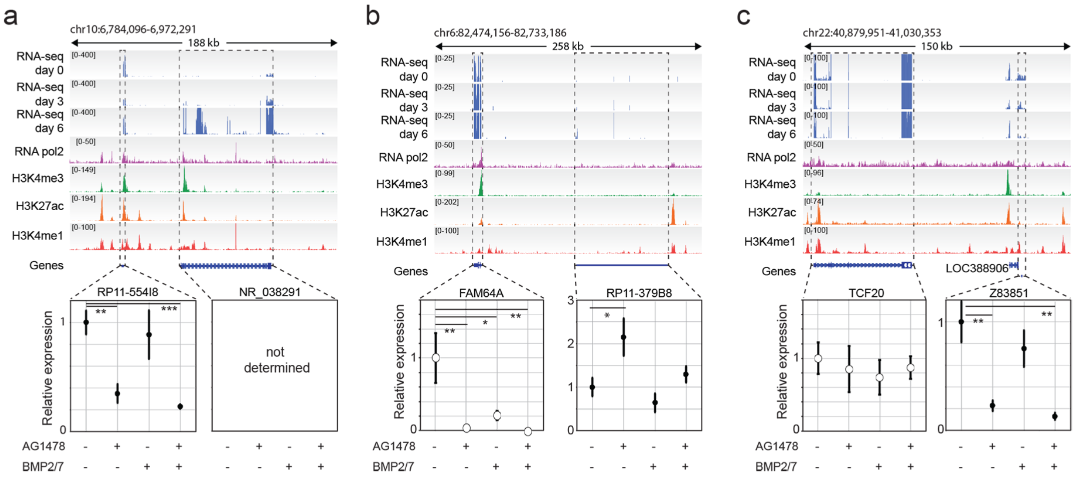

Supplemental figure 1: **Epigenetic landscape surrounding lncRNAs and adjacent genes and their expression upon induction of differentiation.**

a. Upper panel: Publically available data of RNA-sequencing show expression of both transcripts (Kretz, M. *et al*., 2012 (GSE35468)) and histone marks (NHEK tracks, ENCODE). Lower panels: response RP11-554I8 and NR_038291 (mRNA expression data n=3, mean -/+ SD. Statistical significance was assessed using a t-test: * p<0,05; ** p<0,01; *** p<0.001) to induction of differentiation.
b. Upper panel: Publically available data of RNA-sequencing show expression of both transcripts (Kretz, M. *et al*., 2012 (GSE35468)) and histone marks (NHEK tracks, ENCODE). Lower panels: response FAM64A and RP11-379B8 (mRNA expression data n=3, mean -/+ SD. Statistical significance was assessed using a t-test: * p<0,05; ** p<0,01; *** p<0.001) to induction of differentiation.
c. Upper panel: Publically available data of RNA-sequencing show expression of both transcripts (Kretz, M. *et al*., 2012 (GSE35468)) and histone marks (NHEK tracks, ENCODE). Lower panels: response TCF20 and Z83851 (mRNA expression data n=3, mean -/+ SD. Statistical significance was assessed using a t-test: * p<0,05; ** p<0,01; *** p<0.001) to induction of differentiation.

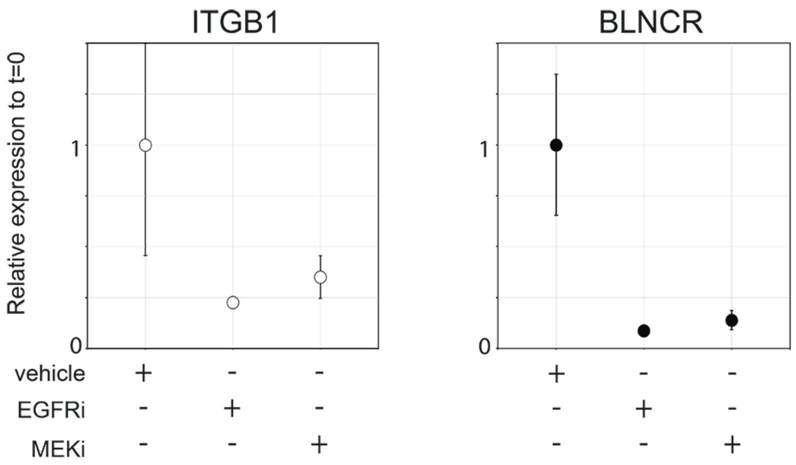

Supplemental figure 2: **Both BLNCR and ITGB1 are downstream of the EGF receptor.** 18s normalized expression of ITGB1 and lncRNA after 24 hours treatment with 10 μM EGFR inhibitor AG1478 or 1 μM MEK inhibitor PD0325901. n=3, t-test indicated no significant differences.

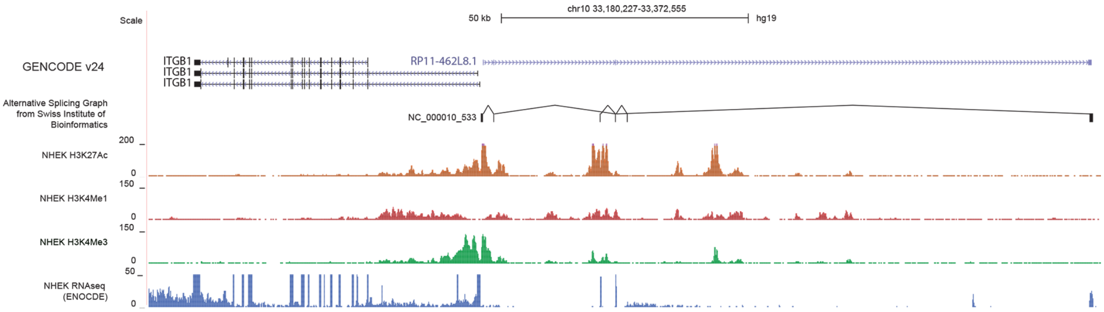

Supplemental figure 3: **BLNCR is an independent transcript that is predicted to be spliced post-transcriptionally.** Tracks include a representation of predicted alternative splicing of BLNCR based on analysis of experimental RNA transcripts (Alternative Splicing Graph from Swiss Institute of Bioinformatics), histone marks H3K27ac, H3K4me1, H3K4me3 and RNA expression in NHEKs

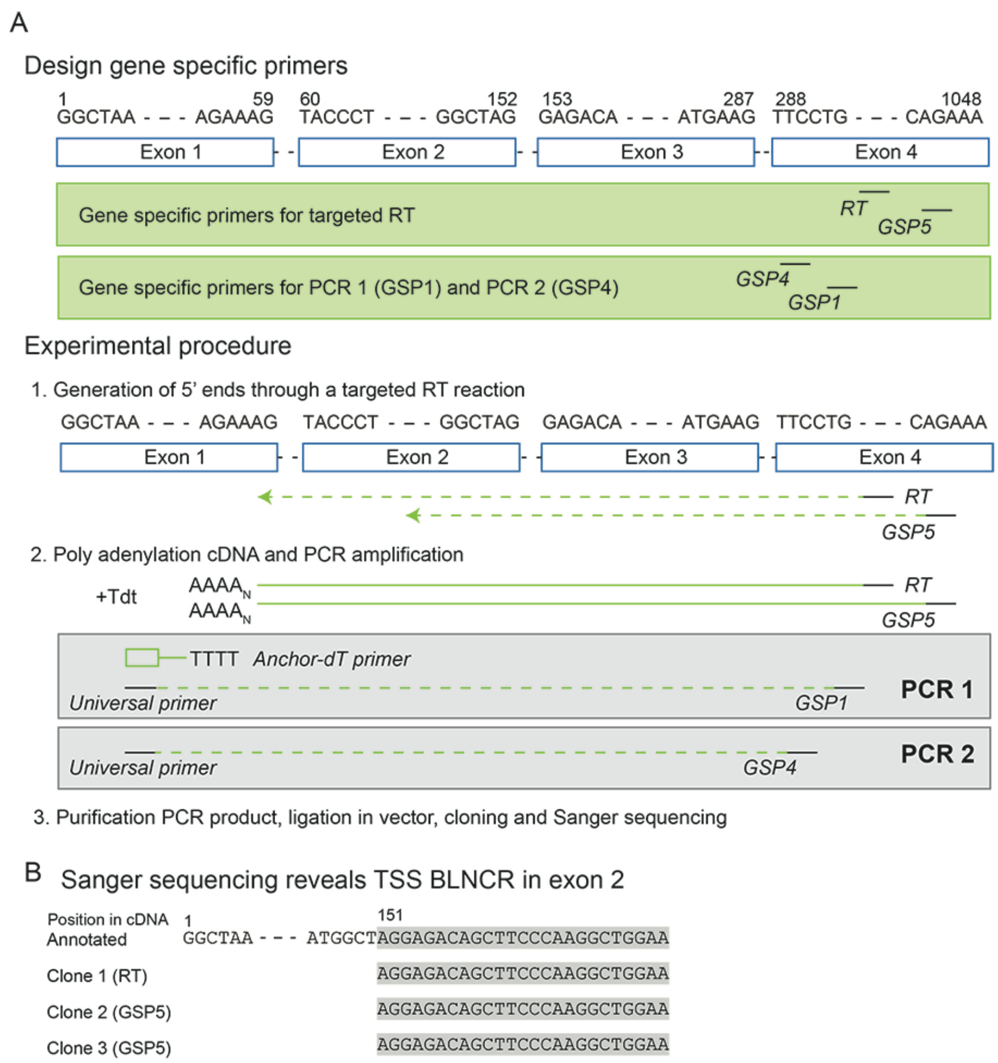

Supplemental figure 4: **5' RACE shows TSS to be located in exon 2.**

- **a.** Schematic overview of the experimental setup and primer combinations.
- **b.** Sanger sequencing results show TSS to be located in exon 2 of the annotated transcript.

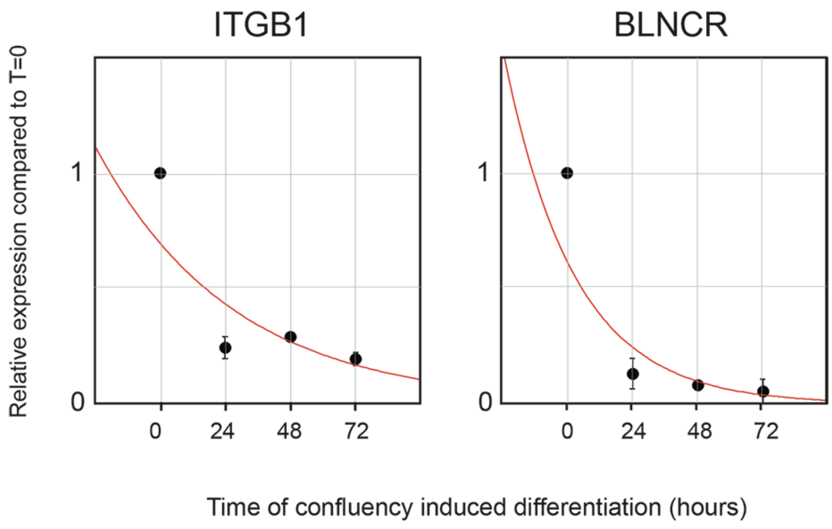

Supplemental figure 5: **Rapid downregulation of BLINCR in confluency induced differentiation assay.**

a. Expression of ITGB1 and BLNCR in PCK19 adult keratinocytes upon confluency induced differentiation and removal of growth factors. n=3, mean -/+ SD.

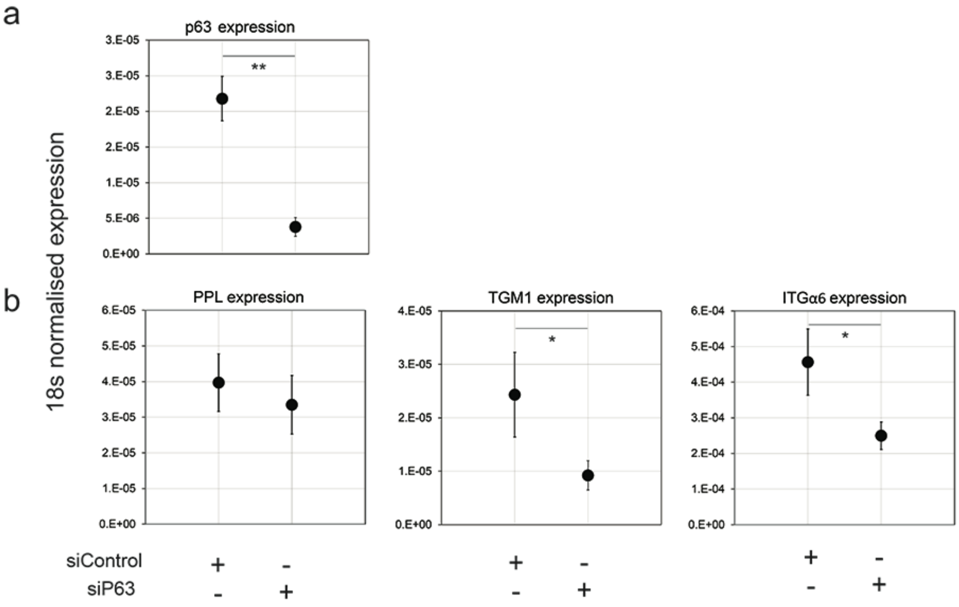

Supplemental figure 6: **Knockdown of p63 affects differentiation and self-renewal markers.**

a. Expression of p63 in the knockdown samples (knockdown of p63 is 82.5%, n=3, mean -/+ SD. Statistical significance was assessed using a t-test: * p<0,05; ** p<0,01; *** p<0.001).
b. Periplakin (PPL), transglutaminase I (TGM1) and integrin α6 (ITGα6) expression in control and p63 knockdown samples (n=3, mean -/+ SD. Statistical significance was assessed using a t-test: * p<0,05; ** p<0,01; *** p<0.001.

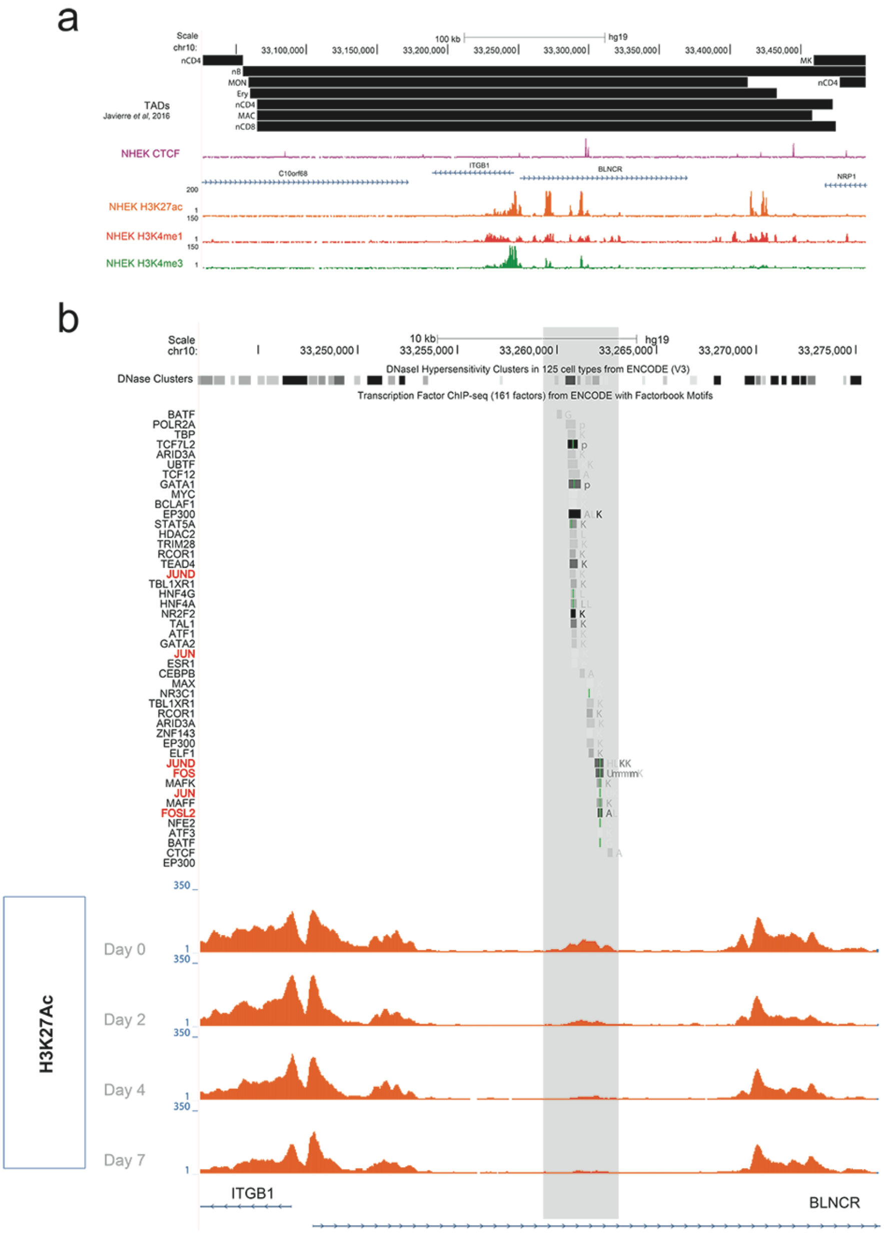

Supplemental figure 7: **Transcription factor binding in ITGB1 and BLNCR containing TAD.**

a. Topology associated domain containing both genes, from Javierre *et al.*, 2016.
b. Transcription Factor ChIP-seq (161 factors) from ENCODE with Factorbook Motifs, AP-1 factors in red and H3K27Ac tracks during differentiation[12].
